# GNRI-stratified enteral nutrition protocol for elderly mechanically ventilated ICU patients: development and preliminary application

**DOI:** 10.3389/fnut.2026.1857582

**Published:** 2026-07-15

**Authors:** Chunxiao Zhang, Yan Zhuang, Jingjing Hong, Huimei Xu

**Affiliations:** The Second People's Hospital of Lianyungang, Lianyungang, Jiangsu, China

**Keywords:** elderly patients, enteral nutrition, geriatric nutritional risk index, mechanical ventilation, nursing

## Abstract

**Objective:**

To develop a clinically feasible enteral nutrition protocol for elderly mechanically ventilated patients in the intensive care unit (ICU) based on the Geriatric Nutritional Risk Index (GNRI) stratification, and to preliminarily evaluate its effects on nutritional indicators, gastrointestinal complications, mechanical ventilation duration, and hospital length of stay.

**Methods:**

Based on systematic literature review and multidisciplinary discussion, a preliminary GNRI-stratified enteral nutrition protocol was formulated. Two rounds of Delphi consultation were conducted among 20 experts to revise and optimize the protocol. A single-center, non-concurrent controlled study was performed in 128 elderly mechanically ventilated patients (63 in the experimental group and 65 in the control group). The experimental group received the standardized GNRI-stratified enteral nutrition protocol, while the control group received conventional nutritional management. The primary outcomes included serum albumin and hemoglobin at 1 week [adjusted by analysis of covariance (ANCOVA) and multivariable linear regression]. Secondary outcomes included enteral nutrition-related complications (analyzed by multivariable logistic regression), mechanical ventilation duration, and hospital length of stay (analyzed by Mann-Whitney *U* test).

**Results:**

The expert authority coefficients were 0.83 and 0.87 in the two rounds, and Kendall's *W* coefficients were 0.299 and 0.335 (both *P* < 0.001), indicating high expert consensus. The final protocol included 5 primary indicators, 14 secondary indicators, and 31 tertiary indicators. After adjustment for baseline levels, the experimental group had significantly higher albumin and hemoglobin at 1 week than the control group (both *P* < 0.001). The overall incidence of enteral nutrition-related complications was significantly lower (7.9% vs. 20.0%, *P* = 0.040), and the adjusted risk of complications was reduced (OR = 0.178, 95%CI: 0.072–0.442, *P* < 0.001). The experimental group also presented significantly shorter mechanical ventilation duration (median 128.0 vs. 172.0 h, *P* = 0.0243) and shorter hospital length of stay (*P* < 0.001).

**Conclusion:**

The GNRI-stratified enteral nutrition protocol developed using the Delphi method is scientific and clinically applicable. In this single-center non-concurrent study, implementation of the protocol was associated with improved nutritional indicators, fewer gastrointestinal complications, shorter mechanical ventilation duration, and shorter hospital stay in elderly mechanically ventilated ICU patients.

## Background

Elderly patients undergoing mechanical ventilation in the intensive care unit (ICU) frequently present in a hypermetabolic state. This hypermetabolic state, driven by underlying disease, severe stress responses, and intestinal dysfunction, predisposes them to a high risk of malnutrition, which in turn severely compromises prognosis and recovery (1, 2). Consistent with this, a high prevalence of malnutrition has been well-documented in this population and is strongly associated with increased complication rates and elevated mortality (3–5). Therefore, the accurate assessment of nutritional risk is paramount for developing effective nutritional support strategies. Malnutrition is strongly associated with a higher incidence of infections and complications, increased mortality, prolonged hospital and ICU length of stay, as well as greater medical economic burden and higher hospitalization costs (6). However, studies establishing enteral nutrition support protocols for elderly mechanically ventilated ICU patients based on Geriatric Nutritional Risk Index (GNRI) stratification remain limited. Most current literature relies on single nutritional screening tools or generic risk scores, lacking individualized, stratified enteral nutrition strategies tailored to distinct GNRI risk categories.

To address this need, the GNRI was introduced by Bouillanne et al. (7) as a tool for predicting 6-month nutritional outcomes in elderly patients admitted to rehabilitation units. The GNRI, which integrates objective measures of height, body weight, and serum albumin, provides a simple and rapid screening method. Its clinical utility has been demonstrated in perioperative nutritional management for elderly patients with gastrointestinal tumors (8). Furthermore, Camiel (9) compared GNRI with the Malnutrition-Inflammation Score (MIS) and showed that GNRI had comparable efficacy in nutritional assessment while offering superior reproducibility. A multicenter cohort study compared the GNRI with the Nutritional Risk in Critically Ill (NUTRIC) score—a tool validated for bedridden, critically ill, unconscious ICU patients—in predicting mortality risk among critically ill patients with acute kidney injury (AKI). The results demonstrated that GNRI showed superior predictive value for mortality compared with the NUTRIC score (10).

However, existing general tools, such as the Nutritional Risk in Critically Ill (NUTRIC) score, may not be optimized for the elderly patient population. Elderly patients undergoing mechanical ventilation often present with age-related physiological changes, comorbidities, and a unique metabolic state, which makes the assessment of their nutritional risk more complex. The Geriatric Nutritional Risk Index (GNRI), introduced as a tool specifically for predicting nutritional outcomes in elderly patients, has demonstrated its clinical utility in perioperative nutritional management for this population. Given its simplicity, effectiveness, and particular applicability in the elderly, the GNRI appears to be a promising tool for risk stratification. Current nutritional support often emphasizes early initiation, but rarely establishes differentiated implementation pathways based on the nutritional risk stratification of elderly patients. Therefore, this study sought to develop and validate the feasibility and effectiveness of a GNRI-stratified enteral nutrition protocol for elderly mechanically ventilated ICU patients.

## Methods

### Study design

#### Establishment and role definition of the research team

A multidisciplinary research team was established, comprising a department director, an attending physician, a head nurse, two part-time research nurses, and a nutritionist. The team members were assigned specific roles: the department director and head nurse oversaw overall quality control, expert selection, and contributed to drafting the preliminary protocol; the attending physician and the nutritionist were involved in the initial drafting of the intervention components; the part-time research nurses were responsible for developing the study protocol, conducting literature searches, designing expert consultation questionnaires, and performing data analysis.

#### Evidence synthesis and literature search

A systematic literature search was conducted to inform the protocol. The search encompassed electronic databases including the Guidelines International Network (GIN), PubMed, Cochrane Library, Embase, Web of Science, UpToDate, China National Knowledge Infrastructure (CNKI), Wanfang Database, Chinese Biomedical Literature Database (CBM), and VIP Chinese Science and Technology Journal Database (VIP). The search strategy employed a combination of subject headings and keywords related to the Geriatric Nutritional Risk Index (GNRI), critically ill elderly patients, and enteral nutrition. Specifically, Chinese search terms included: “老年营养风险指数,” “GNRI,” “重症患者,” “肠内营养,” “营养支持,” “营养评估,” “老年患者,” “营养干预,” and “鼻饲.” Corresponding English terms included: “nutritional risk index,” “GNRI,” “critically ill patients,” “nutritional assessment,” “enteral nutrition,” “elderly patients,” “nutritional intervention.” The search period covered from database inception to September 2024. Inclusion Criteria: Elderly patients (aged ≥60 years) undergoing mechanical ventilation in the intensive care unit (ICU);Studies involving enteral nutrition (EN) assessment, implementation, monitoring, complication prevention, or nutritional risk stratification (including GNRI-related evidence); Clinical practice guidelines, expert consensus statements, evidence summaries, systematic reviews, randomized controlled trials (RCTs), and cohort studies; Full texts published in Chinese or English; Published between January 2010 and September 2024 (to ensure currency of evidence). Exclusion Criteria: Studies focusing on pediatric, non-mechanically ventilated, or non-elderly ICU populations; Case reports, conference abstracts, narrative reviews, duplicate publications.

Following the application of inclusion and exclusion criteria, 14 studies were finally included. The evidence comprised 5 clinical practice guidelines (11–15), 3 evidence summaries (16–18), 2 expert consensus statements (19, 20), 2 systematic reviews (21, 22), 1 randomized controlled trial (23), and 1 cohort study (24). The research team members independently performed quality assessments of the included literature using appropriate critical appraisal tools (e.g., AGREE II for guidelines, Cochrane RoB 2 for RCTs) and extracted relevant evidence. Based on the synthesized evidence, a preliminary draft of the GNRI-stratified enteral nutrition protocol for elderly, mechanically ventilated ICU patients was developed.

#### Expert consultation process

A structured expert consultation was undertaken to refine the draft protocol. The consultation materials consisted of three parts: 1) an introduction to the research background, objectives, and significance; 2) an expert qualification inquiry, collecting basic information (gender, age, years of experience, educational background, and academic qualifications) and self-assessments on their familiarity with the topic and the basis for their judgments; and 3) the core component, the “Draft Enteral Nutrition Protocol for Elderly ICU Patients Undergoing Mechanical Ventilation Based on Geriatric Nutritional Risk Index (GNRI) Grading.”

#### Data collection and analysis

Experts rated the importance of each indicator using a 5-point Likert scale (1 = unimportant, 5 = extremely important). We calculated the expert authority coefficient (derived from self-rated familiarity and judgment basis), mean importance score, coefficient of variation (CV), and full-mark rate for each item. Prespecified criteria were used for indicator retention, revision, or deletion: an indicator was retained if the mean importance score ≥ 4.0, CV < 0.25, and full-mark rate ≥ 30%. Indicators not meeting these criteria were subjected to further evaluation. All qualitative expert comments and suggestions were collected, summarized, and thematically analyzed. The research team then held group discussions to comprehensively determine whether each indicator should be retained, revised, or deleted based on the quantitative criteria and qualitative expert feedback. The protocol was revised accordingly before the next round of consultation.

#### Two-round Delphi consultation

A total of 20 experts were invited to participate in this Delphi consultation. Inclusion criteria for experts were clearly defined as follows: (1) holding senior or associate senior professional title; (2) engaged in critical care medicine, critical care nursing, or clinical nutrition for 10 years or more; (3) having clinical practice or research experience related to enteral nutrition, critical care nutrition, or elderly nutrition. Experts were selected based on their familiarity with the research field and voluntary informed consent.

The experts represented diverse regions across China, including Shanghai, Jiangsu, Beijing, Shandong, Zhejiang, and Sichuan. The draft protocol was distributed electronically via email and WeChat. After the first round of consultation, all quantitative scores and qualitative comments were collected and analyzed, and the research team conducted group discussions to revise the questionnaire accordingly. The revised questionnaire was redistributed for a second round after a 2-week interval. The consultation process was terminated when expert opinions reached consensus, defined as no new substantial suggestions or modifications.

#### Study participants and design

A non-concurrent controlled trial was conducted in the ICU of a tertiary Grade A hospital in Lianyungang, China, from October 2024 to September 2025. Participants were elderly (>60 years) patients receiving mechanical ventilation. Exclusion criteria included: concurrent participation in another interventional study; withdrawal of consent; transfer to another facility; discharge against medical advice.

#### Group allocation and blinding

Patients admitted between October 2024 and March 2025 were assigned to the control group, and those admitted between April and September 2025 comprised the experimental group. A non-concurrent design was employed to minimize contamination between groups. While blinding of participants and clinicians to the nutritional protocol was not feasible due to the nature of the intervention, outcome assessors and data analysts were blinded to group assignment.

#### Sample size calculation

The sample size was calculated for a comparison of two independent means. Using serum albumin as the primary outcome measure, parameters from prior literature (25) were applied to the formula: *n* $=\frac{2\times {\left(Z_{\frac{\alpha }{2}}+Z_{\beta }\right)}^{2}\times \sigma^{2}}{\delta^{2}}$, with a two-sided αof 0.05 and β of 0.10. Based on data from a recent RCT involving critically ill patients with high nutritional risk, the baseline serum albumin was approximately 2.8 g/dL with a pooled standard deviation (σ) of 0.68 g/dL. A mean difference (δ) of 0.4 g/dL between the experimental and control groups was considered clinically significant. The initial calculation indicated that 46 patients were required per arm. Accounting for a 10% attrition rate, the required sample size was 51 patients per group (102 in total). A total of 128 eligible patients were finally enrolled in this study (63 in the experimental group and 65 in the control group) to ensure sufficient statistical power.

## Interventions

### Intervention methods

Patients in the control group underwent nutritional risk screening using the Nutritional Risk Screening 2002 (NRS2002). The attending physicians used a conventional nutritional intervention model to decide whether to initiate enteral nutrition support, as well as the timing and regimen. According to the patient's nutritional risk level and metabolic status, the energy target was typically set at 20–35 kcal/kg/day. For patients at high nutritional risk, the recommended energy target was 30–35 kcal/kg/day. Protein intake was adjusted based on the patient's nutritional risk level and disease status. The general recommended protein intake was 1.2–2.0 g/kg/day, and for patients at high nutritional risk, the protein intake was 1.5–2.0 g/kg/day. Nutritional solutions were administered following the “gradual progression” principle, starting at a low rate and small volume, then gradually increasing after tolerance was achieved. The tube was flushed every 4 h, the head of the bed was kept elevated, and complications were closely monitored. If any abnormality occurred, administration was stopped immediately and the physician was notified.

### Experimental group

The experimental group received a GNRI-stratified enteral nutrition protocol for elderly mechanically ventilated ICU patients (Table 1). Before implementation, all healthcare personnel completed standardized training on the protocol, including nutritional risk assessment, stepwise protocol delivery, and monitoring and management of enteral nutrition-related complications.

**Table 1:** Degree of coordination of expert opinions in the two consultation rounds.

Category	Round 1				Round 2			
	Number of items	Kendall's *W*	*χ^2^* value	*P*-value	Number of items	Kendall's *W*	*χ^2^* value	*P*-value
Primary indicators	5	0.324	25.891	< 0.001	5	0.339	29.091	< 0.001
Secondary indicators	15	0.318	89.126	< 0.001	14	0.357	92.933	< 0.001
Tertiary indicators	23	0.292	138.332	< 0.001	31	0.333	199.845	< 0.001
Total	45	0.299	261.349	< 0.001	50	0.335	328.116	< 0.001

### Outcome measures

(1) Nutritional indicators

In the morning after an overnight fast, 10 mL of venous blood was collected from each patient to measure serum albumin (Alb) and hemoglobin (Hb) levels.

(2) Nutritional complications

The incidence of digestive system complications related to nutritional support (gastric retention, nausea/vomiting, and diarrhea) was recorded and compared between the two groups during the period of nutritional support therapy.

(3) Other indicators

The duration of mechanical ventilation and ICU length of stay were compared between the two groups.

### Data collection and quality control methods

A self-designed general data collection form was used to collect patients' gender, age, body mass index (BMI), APACHE II score, NRS2002 score, and GNRI score. To ensure homogeneity of implementation, the medical team leaders and head nurses provided training to healthcare staff. Department team leaders supervised the implementation of the nutritional protocol and assessed accuracy and complications through medical record reviews. Research team members were responsible for data collection and document retrieval; they received standardized operational training before implementation to ensure accurate data collection methods. Trial data entry was verified by dual personnel to ensure accuracy.

### Statistical methods

SPSS 26.0 (IBM Corp., Armonk, NY, USA) was used. Normality was assessed by Shapiro-Wilk test. Normally distributed data were expressed as mean ± SD and compared by independent *t*-test; non-normally distributed data as median (IQR) and compared by Mann-Whitney *U* test. Categorical variables were analyzed by chi-square or Fisher's exact test. To adjust for baseline imbalances, ANCOVA was used for post-intervention Alb and Hb (with baseline values as covariates), multiple linear regression for 1-week changes in Alb and Hb, and logistic regression for complications. Effect sizes (Cohen's d, Cliff's delta, odds ratio) with 95% confidence intervals were reported. For the Delphi process, expert authority (Cr), coefficient of variation, and Kendall's *W* were calculated. *P* < 0.05 was considered significant.

## Results

### Expert consultation results

A total of 20 experts participated in the consultation. Their mean age was 43.15 ± 4.30 years, and 80% (16/20) were female. Regarding educational attainment, 50% (10/20) held a bachelor's degree, 35% (7/20) a master's degree, and 15% (3/20) a doctoral degree. In terms of professional titles, 30% (6/20) had a senior title, 40% (8/20) a deputy senior title, and 30% (6/20) an intermediate title. The mean years of working experience were 19.30 ± 5.26 years.

In both consultation rounds, 20 questionnaires were distributed and all were returned, giving an effective response rate of 100%. The expert authority coefficients were 0.83 and 0.87 for the first and second rounds, respectively. Kendall's concordance coefficients (*W*) were 0.299 (first round) and 0.335 (second round), with both *P* < 0.001. All items received importance scores above 4 (the predefined threshold for inclusion was ≥4.0), and all coefficients of variation were below 0.25 (Table 1).

### Expert opinion synthesis and protocol revision

Based on the experts' feedback, the following modifications were made to the draft protocol. It was suggested that the secondary indicator “GNRI grading” be revised to “nutritional risk screening.” The item “reassess GNRI every 48 h” was changed to “assess GNRI within 2 h of ICU admission and then every 7 days thereafter.” The indicator “monitor 24-h intake and output” was removed, as it is considered part of routine care for critically ill patients. Additional indicators were added, including “position management (semi-recumbent position when clinically feasible),” “management of abnormal blood glucose,” and “switch to post-pyloric feeding in patients requiring high doses of sedatives/analgesics or presenting with marked abdominal distension.”

The final version of the GNRI-stratified enteral nutrition protocol for elderly ICU patients on mechanical ventilation comprised 5 primary indicators, 14 secondary indicators, and 31 tertiary indicators (Table 2).

**Table 2:** GNRI-stratified enteral nutrition protocol for elderly ICU patients on mechanical ventilation.

Item	Importance (mean ±SD)	Coefficient of variation
**I. Nutritional assessment and monitoring**	4.95 ± 0.22	0.045
I-1. Nutritional risk screening	4.50 ± 0.51	0.114
I-1-1. Assessors: ICU physician, ICU nurse, dietitian	4.40 ± 0.50	0.114
I-1-2. Assess patient's primary disease, hemodynamic status, contraindications, etc.	4.40 ± 0.50	0.114
I-1-3. Use GNRI tool. Formula: GNRI = 14.89 × serum albumin (g/dL) + 41.7 × (actual body weight/ideal body weight), with the weight ratio capped at 1 if actual body weight > ideal body weight. Ideal body weight (kg) = height (m) × height (m) × 22.	4.95 ± 0.22	0.045
I-1-4. Grading criteria: low risk (≥98), moderate risk (92–97), high risk (< 92)	4.95 ± 0.22	0.045
I-2. Dynamic monitoring	4.40 ± 0.51	0.114
I-2-1. Assess within 2 h of ICU admission, then every 7 days thereafter	4.40 ± 0.50	0.114
**II. Timing of nutritional support initiation**	4.45 ± 0.51	0.115
II-1. Initiation timing	4.40 ± 0.50	0.114
II-1-1. In mechanically ventilated patients with essentially stable hemodynamics and no contraindications to enteral nutrition (e.g., intestinal obstruction, perforation, severe peritonitis), initiate enteral nutrition within 24 h (at most 48 h) after ICU admission	4.90 ± 0.31	0.063
II-1-2. Contraindications: intestinal obstruction, severe diarrhea, AGI grade IV, etc.	4.40 ± 0.50	0.114
**III. Nutritional goal setting**	4.95 ± 0.22	0.045
III-1. Energy and protein targets	5.00 ± 0.00	0
III-1-1. Low risk: 25–30 kcal/(kg·d), protein 1.2–1.5 g/(kg·d)	4.95 ± 0.22	0.045
III-1-2. Moderate risk: 30–35 kcal/(kg·d), protein 1.5–2.0 g/(kg·d)	4.95 ± 0.22	0.045
III-1-3. High risk: 35 kcal/(kg·d), protein ≥2.0 g/(kg·d), may be increased based on clinical conditions (e.g., continuous renal replacement therapy)	4.40 ± 0.50	0.114
**IV. Nutrition implementation strategies**	4.85 ± 0.37	0.076
IV-1. Feeding route	4.90 ± 0.31	0.063
IV-1-1. ICU physician assesses the patient; nasogastric tube is preferred	4.40 ± 0.50	0.114
IV-1-2. If gastric residual volume ≥500 mL, switch to post-pyloric feeding	4.40 ± 0.50	0.114
IV-1-3. When patients require high doses of sedatives/analgesics or present with marked abdominal distension, change to post-pyloric feeding	4.40 ± 0.50	0.114
IV-2. Formula selection	4.40 ± 0.50	0.114
IV-2-1. Low risk: standard whole-protein formula	4.95 ± 0.22	0.045
IV-2-2. Moderate/high risk: short-peptide formula with dietary fiber	4.95 ± 0.22	0.045
IV-2-3. With concomitant renal dysfunction: low-phosphorus, low-potassium formula	4.40 ± 0.50	0.114
IV-3. Infusion method	5.00 ± 0.00	0
IV-3-1. Continuous infusion via enteral nutrition pump (starting at 20–50 mL/h)	4.95 ± 0.22	0.045
IV-3-2. For high-risk patients, use “stepwise incremental method” (increase by 25% per day) to reach target feeding volume around day 4	4.40 ± 0.50	0.114
IV-4. Position management	5.00 ± 0.00	0
IV-4-1. Semi-recumbent position (30 °-45 °) is recommended when clinically feasible	4.40 ± 0.50	0.114
IV-4-2. During prone positioning, avoid abdominal compression and keep head of bed elevated to reduce intra-abdominal pressure	4.95 ± 0.22	0.045
**V. Complication management**	4.40 ± 0.50	0.114
V-1. Assessment method	5.00 ± 0.00	0
V-1-1. Use an enteral nutrition tolerance assessment tool, including abdominal distension and/or pain, diarrhea, nausea and/or vomiting, and bowel sounds	4.95 ± 0.22	0.045
V-2. Abdominal distension and/or pain	4.40 ± 0.50	0.114
V-2-1. Reduce lactose/fat content, combine with probiotics (e.g., *Bifidobacterium*)	4.45 ± 0.51	0.115
V-2-2. If intra-abdominal pressure exceeds 20 mmHg, temporarily suspend enteral nutrition and re-measure after 4 h; if still >20 mmHg, switch to post-pyloric feeding as ordered	4.40 ± 0.50	0.114
V-3. Diarrhea management	4.40 ± 0.50	0.114
V-3-1. For enteral nutrition-related diarrhea, use a dedicated warmer to adjust formula temperature to near body temperature	4.45 ± 0.51	0.115
V-3-2. Do not immediately stop enteral nutrition when diarrhea occurs. After consulting the physician, slow the infusion rate, reduce total formula volume, or change the formula. If feasible, add soluble fiber (20 g/L)	4.40 ± 0.50	0.114
V-4. Aspiration management	5.00 ± 0.00	0
V-4-1. For patients at high risk of aspiration or with a history of reflux/aspiration, choose post-pyloric feeding	4.95 ± 0.22	0.045
V-4-2. Monitor cuff pressure every 4 h and maintain it at 25–30 cmH_2_O	4.90 ± 0.31	0.063
V-4-3. Regularly assess airway secretions and clear them promptly; subglottic suctioning can reduce aspiration risk	4.95 ± 0.22	0.045
V-5. Nausea and/or vomiting	4.40 ± 0.50	0.114
V-5-1. Use gradual feeding to reduce gastrointestinal irritation; dynamically monitor gastric residual volume by bedside ultrasound	5.00 ± 0.00	0.000
V-5-2. If vomiting occurs, turn the patient's head to one side to avoid aspiration, observe and record the color, nature, and volume of vomitus	4.40 ± 0.50	0.114
V-6. Abnormal blood glucose	4.50 ± 0.61	0.135
V-6-1. Monitor blood glucose (target 7.8–10.0 mmol/L). When blood glucose >10.0 mmol/L, administer insulin infusion as ordered and adjust dose based on blood glucose results; insulin therapy if necessary	4.50 ± 0.61	0.135

### Application results

A total of 128 patients were enrolled, including 63 in the experimental group and 65 in the control group.

Baseline characteristics including age, sex, BMI, APACHE II score, NRS-2002 score, GNRI score, ventilatory parameters, and inflammatory indicators were generally comparable between the two groups (all *P* > 0.05; Table 3).

**Table 3:** Comparison of baseline characteristics between the two groups (*n* = 128).

Characteristic	Category	Experimental group (*n* = 63)	Control group (*n* = 65)	Statistic (*χ^2^*/*t*/*Z*)	*P*-value
Sex	Male	31 (49.2)	36 (55.4)	0.033	0.855
Age (years)		73.58 ± 7.00	74.83 ± 7.66	0.933	0.353
BMI (kg/m^2^)	< 18.5	1 (1.6)	1 (1.5)	0.578	0.749
	18.5 ≤ BMI < 25.0	56 (88.9)	55 (84.6)		
	≥25.0	6 (9.5)	9 (13.8)		
APACHE-II score		22.76 ±8.24	21.94 ±7.35	−0.596	0.552
SOFA score		11.33 ± 3.25	10.98 ±3.39	−0.594	0.554
NRS score		5.78 ± 1.01	6.07 ± 1.10	1.554	0.123
GNRI score		90.36 ± 5.67	91.57 ± 5.59	−1.175	0.243
Admission diagnosis				0.44	0.51
Respiratory disease		13 (21.7)	16 (26.7)		
Sepsis/septic shock		7 (11.7)	5 (8.3)		
Cardiovascular disease		8 (13.3)	10 (16.7)		
Neurological disease		11 (18.3)	7 (11.7)		
Surgery		21 (35.0)	22 (36.7)		
Comorbidities on admission				1.517	0.678
COPD		3 (4.8%)	1 (1.5%)		
Hypertension		5 (7.9%)	4 (6.2%)		
Diabetes mellitus		6 (9.5%)	5 (7.7%)		
Pre-ICU medications				4.095	0.251
Antibiotics only		2 (6.1%)	0 (0.0%)		
Vasopressors only		17 (51.5%)	17 (54.8%)		
Antibiotics +vasopressors		12 (36.4%)	14 (45.2%)		
Mechanical ventilation duration					
Pressure support (PS, cmH_2_O)		9.75 ± 2.36	9.71 ± 2.34	−0.092	0.927
PEEP (cmH_2_O)		5.76 ± 1.71	5.91 ± 1.59	0.499	0.618
PaO_2_/FiO_2_ (mmHg)		209.48 ± 49.75	217.93 ±55.22	0.909	0.365
Respiratory rate (breaths/min)		21.29 ± 2.94	21.52 ± 2.74	0.472	0.637
Minute ventilation (L/min)		7.71 ± 0.45	7.60 ± 0.49	−1.217	0.226
White blood cell count ( × 10?/L)		11.79 ± 4.55	11.59 ± 4.61	−0.243	0.808
Neutrophil (%)		84.03 ± 6.57	84.74 ± 7.62	0.567	0.572
Blood urea nitrogen (mmol/L)		8.67 ± 5.91	7.42 ± 3.91	−1.404	0.163
Creatinine (μmol/L)		79.56 ± 59.38	72.69 ± 50.16	−0.705	0.482
C-reactive protein (mg/L)		47.21 ± 35.09	58.26 ± 57.05	1.324	0.188
Procalcitonin (ng/mL)		1.77 ± 5.38	2.38 ± 12.50	0.363	0.717

APACHE-II, Acute Physiology and Chronic Health Evaluation II; BMI, body mass index; GNRI, Geriatric Nutritional Risk Index; NRS, Nutritional Risk Screening 2002.

At baseline, serum albumin (Alb) and hemoglobin (Hb) levels were similar between the two groups (both *P* > 0.05). After intervention, Alb and Hb were significantly increased in both groups (both *P* < 0.05), with significantly greater improvements in the experimental group (both *P* < 0.05; Tables 4, 5).

**Table 4:** Analysis of covariance (ANCOVA) for post-treatment outcomes adjusted for baseline values.

Dependent variable/source	SS	df	MS	*F*	*P*
One-week albumin					
Baseline albumin	24.93	1	24.93	15.4	0.0001
Group	227.69	1	227.69	140.61	< 0.0001
Error	202.41	125	1.62		
One-week hemoglobin					
Baseline hemoglobin	1,663.92	1	1,663.92	15.2	0.0002
Group	20,886.16	1	20,886.16	190.77	< 0.0001
Error	13,685.13	125	109.48		

SS, sum of squares; MS, mean square (SS/df). *F* values were computed as the ratio of the respective MS to the error MS.

**Table 5:** Multivariable linear regression of 1-week changes in nutritional indicators.

Dependent variable	Independent variable	Coefficient (*B*)	SE	*t*	*P*	95% CI
Change in albumin (g/L)	Intercept	11.9719	2.1361	5.6045	< 0.0001	7.7429~16.2009
	Group (Experimental group)	0.7809	0.2234	3.4954	0.0007	0.3386~1.2231
	Age	−0.0167	0.0158	−1.0556	0.2933	−0.0479~0.0146
	Sex	−0.2087	0.2287	−0.9125	0.3633	−0.6614~0.2441
	SOFA score	0.1257	0.0528	2.3789	0.0189	0.0211~0.2303
	APACHE II score	−0.0380	0.0226	−1.6845	0.0947	−0.0827~0.0067
	Baseline albumin	−0.3153	0.0572	−5.5139	< 0.0001	−0.4286~−0.2021
Change in hemoglobin (g/L)	Intercept	31.0046	11.1199	2.7882	0.0062	8.9897~53.0194
	Group (Experimental group)	8.995	1.8158	4.9538	< 0.0001	5.4002~12.5899
	Age	−0.1333	0.1293	−1.0307	0.3047	−0.3892~0.1227
	Sex	−0.6940	1.8234	−0.3806	0.7042	−4.3039~2.9160
	SOFA score	1.3428	0.429	3.1299	0.0022	0.4935~2.1922
	APACHE II score	−0.4762	0.1824	−2.6111	0.0102	−0.8373~−0.1151
	Baseline hemoglobin	−0.2026	0.056	−3.6148	0.0004	−0.3135~−0.0916

The overall incidence of enteral nutrition–related complications was significantly lower in the experimental group than in the control group (*P* < 0.05; Tables 6, 7).

**Table 6:** Comparison of enteral nutrition complications between the two groups.

Group	*n*	Diarrhea	Abdominal distension and/or pain	Nausea and/or vomiting	Aspiration	Total complications
Experimental group	63	1 (1.6%)	2 (3.2%)	1 (1.6%)	1 (1.6%)	5 (7.9%)
Control group	65	4 (6.2%)	4 (6.2%)	3 (4.6%)	2 (3.1%)	13 (20.0%)
*χ^2^*/Fisher						4.231
*P*-value						0.040

**Table 7:** Multivariate logistic regression analysis for risk factors of enteral nutrition complications.

Variable	*B*	SE	*Z*	*P*-value	OR	95% CI
Intercept	3.2452	4.0382	0.8036	0.4216	25.6673	0.0094–70262.7387
Group (Experimental group)	−1.7261	0.4645	−3.7162	0.0002	0.178	0.0716–0.4423
Age	0.0312	0.0304	1.0256	0.3051	1.0317	0.9720–1.0951
Sex	0.4852	0.4408	1.1006	0.2711	1.6244	0.6847–3.8544
SOFA score	−0.1022	0.1019	−1.0028	0.316	0.9029	0.7395–1.1024
APACHE II score	0.0157	0.0436	0.361	0.7181	1.0159	0.9326–1.1065
Baseline albumin	−0.1602	0.1107	−1.4465	0.148	0.852	0.6858–1.0585

The experimental group also showed shorter mechanical ventilation duration and ICU length of stay compared with the control group (both *P* < 0.05; Table 8).

**Table 8:** Comparison of length of hospital stay and mechanical ventilation duration between groups (median, non-parametric test).

Index	Control group median (IQR)	Experimental group median (IQR)	Mann-Whitney *U*	*P*-value	Cliff's delta (95% CI)
Length of hospital stay, d	18.0 (15.0–23.0)	13.0 (10.0~15.5)	3,168.5	< 0.001	0.5475 (0.3812~0.6960)
Mechanical ventilation duration, h	172.0 (124.0–240.0)	128.0 (104.0–180.0)	2,520.5	0.0243	0.2310 (0.0302–0.4319)

In-hospital mortality was analyzed as a separate secondary outcome. During the ICU stay, 3 of 63 patients (4.8%) died in the experimental group and 5 of 65 patients (7.7%) died in the control group. Fisher's exact test showed no statistically significant difference between the two groups (*P* = 0.489).

## Discussion

### The GNRI-stratified enteral nutrition protocol for elderly ICU patients on mechanical ventilation is scientifically sound and acceptable expert consensus and feasibility

The present study developed a GNRI-stratified enteral nutrition protocol for elderly ICU patients on mechanical ventilation based on an extensive literature review and a two-round Delphi expert consultation. The Delphi method leverages collective expert judgment to overcome the limitations of individual decision-making, thereby enhancing the content validity and clinical feasibility of the resulting protocol (26, 27). To ensure the credibility of the consensus, we invited 20 experts from critical care medicine, nursing, and clinical nutrition across multiple regions in China. Their high level of expertise was reflected by the fact that 70% held senior or associate senior titles and 50% held a master's degree or higher. The questionnaire response rate was 100% in both rounds, and many experts provided detailed constructive comments, indicating strong engagement.

The scientific robustness of the protocol is supported by three quantitative indicators. First, the expert authority coefficients (Cr) were 0.83 and 0.87, well above the generally accepted threshold of 0.70, confirming that the panel possessed sufficient knowledge and experience to make credible judgments. Second, after two rounds, all protocol items received mean importance scores >4 (out of 5) and coefficients of variation < 0.25, demonstrating that the experts consistently rated each component as necessary and appropriate. Third, Kendall's concordance coefficients (*W*) were 0.299 in the first round and increased to 0.335 in the second round (both *P* < 0.001), indicating that panel opinions converged after iterative feedback. Collectively, these findings confirm that the final protocol—comprising 5 primary, 14 secondary, and 31 tertiary indicators—has strong content validity, broad expert endorsement, and practical applicability for clinical nursing practice.

### Implementation of the protocol was associated with improvements in patients' nutritional status

Gassel et al. (28) reported that during the acute phase of illness, critically ill patients lose approximately 12–16 g of protein per day, and the loss can reach up to 30 g. When malnutrition occurs, protein intake is insufficient and hepatic synthesis is impaired, leading to decreased albumin production (29, 30). Thus, adequate nutritional support is crucial for these patients. The GNRI, which integrates serum albumin and body weight changes, provides a practical tool for nutritional risk assessment in elderly critically ill patients. In the present study, after adjusting for baseline values and potential confounders (age, sex, SOFA, APACHE II), the experimental group showed significantly greater improvements in both serum albumin and hemoglobin compared with the control group (both *P* < 0.05, Tables 4, 5). Multivariable linear regression further confirmed that group assignment was independently associated with 1-week changes in albumin (*B* = 0.78, 95% CI: 0.34–1.22, *P* = 0.0007) and hemoglobin (*B* = 8.99, 95% CI: 5.40–12.59, *P* < 0.0001), after controlling for baseline levels, disease severity, and demographic factors (Table 5).

However, several limitations must be acknowledged when interpreting these findings. Serum albumin is an acute-phase reactant that is influenced by systemic inflammation, fluid balance, capillary leak, and liver synthetic function, rather than being a pure marker of nutritional status (31, 32). Therefore, the observed improvement in albumin in the experimental group may partially reflect better disease control or reduced inflammation, rather than solely nutritional repletion. Similarly, hemoglobin levels can be affected by blood transfusions, occult bleeding, hemodilution, and other non-nutritional factors, and the modest increase observed does not necessarily indicate correction of anemia. Nevertheless, the consistency of findings across unadjusted, ANCOVA, and regression analyses—and the fact that SOFA score was independently associated with albumin change (*B* = 0.13, *P* = 0.019, Table 5)—suggests that both nutritional support and overall clinical improvement contributed to the observed changes. Based on GNRI grading, the protocol established graded energy and protein targets to deliver individualized feeding strategies. This demand-based model may better address the hypermetabolic needs of critically ill elderly patients and was associated with improved biochemical outcomes, consistent with findings by Lai et al. (33) who reported that GNRI is a reliable and reproducible nutritional assessment tool.

### Individualized nutritional protocol was associated with fewer complications and improved feeding tolerance

In this study, the overall incidence of enteral nutrition–related complications was significantly lower in the experimental group than in the control group (χ^2^ = 4.231, *P* = 0.040; Table 6). After adjusting for age, sex, SOFA, APACHE II, and baseline albumin, multivariable logistic regression confirmed that the experimental group had a substantially lower risk of any complication (adjusted OR = 0.178, 95% CI: 0.072–0.442, *P* = 0.0002; Table 7). The most common individual complications were diarrhea (experimental: 1.6% vs. control: 6.2%), abdominal distension and/or pain (3.2% vs. 6.2%), and nausea/vomiting (1.6% vs. 4.6%), all showing numerical trends favoring the experimental group (Table 6). These findings suggest that the GNRI-stratified protocol was associated with improved gastrointestinal tolerance.

Several factors may explain this association. For moderate- or high-risk patients (GNRI < 98), the protocol recommended short-peptide formulas containing dietary fiber, which require less digestive breakdown and may reduce the risk of diarrhea and abdominal distension compared with standard whole-protein formulas (34–36). The “stepwise incremental” infusion method—starting at 20–50 mL/h and increasing by 25% of the target volume per day—allows gradual intestinal adaptation, potentially minimizing osmotic diarrhea and gastric discomfort. In addition, position management (semi-recumbent positioning), routine cuff pressure monitoring, and early switching to post-pyloric feeding in selected patients may have contributed to lower aspiration risk and better overall tolerance. These integrated measures likely acted synergistically to support more reliable and tolerable enteral nutrition delivery. To minimize potential bias associated with the non-concurrent design, all nursing staff received standardized training and strictly followed the unified protocol, ensuring consistent implementation across the two study periods.

### Implementation of the GNRI-stratified protocol was associated with shorter mechanical ventilation duration and ICU length of stay

In this study, the experimental group showed a significantly shorter mechanical ventilation duration [median 128.0 h vs. 172.0 hours, Mann-Whitney *U* = 2,520.5, *P* = 0.0243, Cliff's delta = 0.231 (95% CI: 0.030–0.432)] and a significantly shorter total length of hospital stay (Table 8).

By integrating serum albumin and the actual/ideal body weight ratio, GNRI enables precise risk stratification. For high-risk patients (GNRI < 92), the protocol provided enhanced protein delivery [≥2.0 g/(kg·d)] and energy support [35 kcal/(kg·d)], which may help preserve respiratory muscle mass and diaphragmatic function, thereby facilitating weaning (37, 38). However, such high targets carry potential risks, including overfeeding, refeeding syndrome, and glycemic instability. To mitigate these risks, the protocol incorporated: (1) a stepwise incremental infusion method (starting at 20–50 mL/h, increasing by 25% daily, reaching full target around day 4); (2) blood glucose monitoring with a target of 7.8–10.0 mmol/L and insulin therapy when >10.0 mmol/L; and (3) daily assessment of electrolytes (especially phosphate, potassium, and magnesium) to detect refeeding syndrome early. No cases of refeeding syndrome or severe hyperglycemia (>13.9 mmol/L) were observed in either group.

In summary, through a closed-loop management system of “accurate assessment—risk stratification—goal-directed intervention,” the GNRI-stratified protocol was associated with shorter mechanical ventilation duration and ICU stay. Nevertheless, residual confounding cannot be excluded, and future randomized controlled trials are needed to confirm causality.

Mortality was comparable between the two groups (4.8% vs. 7.7%, *P* = 0.489). This finding suggests that the GNRI-stratified protocol was not associated with increased mortality risk in this vulnerable population. However, given the small sample size, the study was not powered to detect mortality differences, and the observed numerical trend warrants cautious interpretation in future larger-scale trials.

## Conclusion

This study systematically integrated the GNRI nutritional assessment into the entire enteral nutrition management process for elderly patients on mechanical ventilation in the ICU, forming a structured protocol encompassing assessment, goal setting, implementation strategies, and complication management. The protocol was developed through a rigorous Delphi expert consultation process, ensuring its scientific validity and clinical applicability. However, given the single-center, non-concurrent controlled design, the findings should be interpreted with caution, and the protocol's effectiveness requires further validation in multicenter, randomized controlled trials.

### Limitations

It was a single-center study with a relatively limited sample size, which may affect the generalizability of the results. Future research should involve multi-center, large-sample randomized controlled trials (RCTs) to further validate the effectiveness of the protocol.

## Data Availability

The raw data supporting the conclusions of this article will be made available by the authors, without undue reservation.
